# Targeting vulnerable populations: a synthetic review on alcohol use and risky sexual behaviour among migrant populations

**DOI:** 10.1186/s12981-016-0117-8

**Published:** 2016-09-22

**Authors:** Emilia Maria Vaz Martins-Fonteyn, Nina Sommerland, Herman Meulemans, Olivier Degomme, Ines Raimundo, Edwin Wouters

**Affiliations:** 1 Department of Sociology, Research Centre for Longitudinal and Life Course Studies, University of Antwerp, University of Antwerp City Campus, Prinsstraat 13, Antwerp, Belgium; 2 International Centre for Reproductive Health, Ghent University, Ghent, Belgium; 3 Faculty of Social Sciences, University Eduardo Mondlane, Maputo, Mozambique; 4 Linde, 10, 1840 Londerzeel, Belgium

**Keywords:** Migrant populations, Alcohol use, Risky sexual behaviour

## Abstract

**Background:**

Research has demonstrated a link between alcohol use and risky sexual behaviour among different types of migrant populations. Therefore, research investigating risk factors associated with alcohol consumption among them is a public health priority. This review aimed to explore the intersection between migration, alcohol consumption and risky sexual behaviour.

**Methods:**

This article is a synthetic review of empirical studies on the association of alcohol and high-risk sexual behaviour among different types of the migrant populations, focusing on measurable outcomes generated from quantitative data. A descriptive analysis generated from global and situational studies was used to interpret the reviewed research and to discuss critically the factors that drive migrants to engage in alcohol consumption and high-risk behaviour.

**Results:**

This review found out that there is a significant and positive association between global and situational alcohol use and several outcomes of risky sexual behaviour among different types of migrant populations. This association was however mainly observed at high quantities and frequencies of alcohol use, mainly among male migrants, and was often tied to a specific situation or context, for instance the type of sexual partner, the level of mobility and to environmental factors such as living arrangements and entertainment venues.

**Conclusions:**

The study supports previous research that alcohol use is associated with risky sexual behaviour among different types of migrant populations. Therefore, future interventions should target mobile, male migrant heavy drinkers. Additional research is needed using more event-level and longitudinal methodologies that overcome prior methodological limitations.

## Background

The existing theory on the relationship between alcohol consumption and risky sexual behaviour posits that high levels of consumption lead to decreased fear of negative consequences of high risk sexual behaviour [1]. It also shows alcohol consumption has a wide range of consequences in terms of health behaviour and health outcomes, especially when combined with risky sexual behaviour [2–5].

For instance, both acute and chronic alcohol use, as well as the venues where people consume alcohol, can increase the likelihood of risky sexual behaviour. This would include engaging in commercial sex and having multiple sex partners (MSP). These behaviours influence the incidence of HIV infection and other Sexually Transmitted Infections (STIs) [6].

A recent line of research is adding a new stratum to the link between alcohol use and risky sexual behaviour by exploring links among a specific and vulnerable population group such as migrants. On the one hand, ample research has linked migrant populations to higher alcohol consumption than the general population [7]. Additionally, migration is increasingly referred to as a significant risk factor for STIs such as HIV [8].

Further, alcohol use behaviours among migrant populations are complex and likely influenced by multiple factors. These include social norms and mental health [9], as well as patterns of migratory process [10].

Since the link between alcohol consumption and risky sexual behaviour has been widely explored in the literature, the aim of this review is to identify the connection between migration, alcohol consumption and risky sexual behaviour. Therefore, the following questions were posed:

Are migrants who consume alcohol more likely to engage in sexual risk behaviour than those who do not? Are alcohol-related sexual risks elevated for the sub-set of migrants than non-migrants? What are the factors among migrants affecting alcohol-related sexual risks?

The better understanding of the association of alcohol use and risky sexual behaviour among migrant populations will assist in developing effective multilevel approaches to mitigate the effects of this combination of risk factors.

## Methods

### Eligibility criteria

A comprehensive literature search was conducted to identify articles meeting the following criteria: empirical articles, measuring the association between alcohol use and an outcome of risky sexual behaviour among migrant populations. Preferred Reporting Items for Systematic Reviews and Meta-Analyses (PRISMA) guidelines were followed. To best assess the influence between alcohol use and risky sexual behaviour, we incorporated quantitative studies. This method was chosen because it is better suited to assess more precise estimates of the group studied. Conversely, qualitative studies are better suited to answering “why” and “how” questions.

In order to evaluate relevant literature, PubMed was searched for English language articles using the following key words: “risk, sexual, behaviour”, OR “condom use” OR “multiple partners” OR “sexual transmitted infection STI”, AND “alcohol use” OR “abuse”, AND “migrant people” OR “population”.

From the search process, 7755 articles reported on the period from January 2003 to December 2015 and these were included in the study selection process. After inclusion of the filters on the subjects “AIDS”, species “humans” and text availability “full text” in the search process, 343 texts were included in the screening process.

Three steps were applied. In the first phase, the authors independently reviewed all titles of the 343 identified research papers. Of the remaining 137 articles, abstracts were assessed. In the third and final phase, the authors retrieved and reviewed the 22 full-length papers. This enabled our researchers to screen them according to requirements of content. The criteria included providing relevant information concerning the relationship between alcohol abuse and risky sexual behaviour among migrant populations.

As well, quality of the chosen methodology was examined. Questions to be answered were:

1. Are the study design, data collection, data collection methods, sampling strategy and analytical approach appropriate?
2. Is the context described sufficiently, and is the range of missing data acceptable?

In accordance with the methodological requirements of systematic review as specified by Mcrae et al. [11] and Bilotta et al. [12], 19 articles met the inclusion criteria. Additionally, given the first author’s extensive prior work on this topic, three articles from past searches were also included.

After excluding articles not meeting the inclusion criteria, this review identified 22 published articles.

### Methodology used to relate alcohol-related sexual risks

This review sought all studies relating to global, situational and event-level-studies of alcohol use linked to any kind of self-reported risky sexual behaviour, among male and female migrant populations. Global studies queries the frequency and quantity of alcohol use as well as the density of drinking establishments in a specified area and correlate with one or more outcomes of risky sexual behaviour.

Some measurements employed in global association studies are the Alcohol Use Identification Test (AUDIT) and binge drinking—more than five drinks in a single drinking occasion. Situational measurements looking into alcohol consumption or being under influence during participants’ most recent engagement in sexual intercourse, and finally event-level-studies provide more detailed information regarding specific sexual acts, in which alcohol was consumed [13].

Outcomes of risky sexual behaviour include: unprotected sex, number and type of sexual partners, commercial and premarital sex, STI, and HIV infections.

## Results

Migration and mobility among a population are operationalised differently amongst the studies reviewed. For a better overview, three types of migrant populations were specified in the included studies. The first and most commonly studied population with regards to alcohol consumption and risky sexual behaviour were migrant labourers. Many of these could be defined as circular migrants. Common denominators for these populations are they stem from rural surroundings and periodically migrate to cities for economic opportunities. During times when they do not need to work, they typically return to their rural homes and their family/spouses [14].

The second category found in the literature is migrant sex workers. The main reason to distinguish this group from other migrant workers is they do not engage in the same circular pattern of work. As well, they often face a singular kind of vulnerability due to the nature of their occupation.

To the third category belong populations who have permanently migrated to a new country or city. The reason why these populations are studied is that several populations have been discovered to have higher rates of alcohol consumption and STI’s than the general population [15].

### Characteristics of the sample

Included in this review are 14 studies of migrant labourers, 2 studies of migrant sex workers and 9 studies of stationary immigrants. The majority, 19 of the populations studied, were male migrant populations. However, few studies focused exclusively upon female migrants and only one study looked at both genders. Overall, the age of the migrant population ranges from 13 to 79 years old.

The majority of articles were found in the USA [7], six in China, three in India and two in Russia, one in Namibia, one in South Africa, one in Bangladesh, and one in Ethiopia. This review found articles presenting very large sample sizes, mostly extracted from large-scale community based household surveys. These included Gupta et al. [10] (*N* = 174.365) and large database from national surveys, e.g. Verma et al. [16] (*N* = 7.602); four studies presented samples from >5.000 to >1000. The majority of the studies (60 %) present sample sizes around >500 to >100.

This review identified 22 quantitative studies, 19 cross-sectional, 2 longitudinal, and 1 ecological study, measuring alcohol use and any outcome of risky sexual behaviour among several types of male and female migrant populations. 19 studies used global alcohol measurements and 3 studies used situational measurements.

### Alcohol use and unprotected sex

Global and situational studies presented in this review found a positive association between alcohol use and unprotected sex among migrant labourer population independent of being male or female [17]. Several studies revealed there was a link between alcohol use and non-use of condoms during sex with paid sex workers [16, 18, 19]. However Organista and Kubo [20] did not find any significant associations between alcohol use and unprotected sex with sex workers.

The migrant Female Sex Workers (FSWs) group also shows inconsistent condom use with paid and non-paid partners both significantly higher if they consumed alcohol prior to sex than when they did not [16]. The same result was presented by Wong et al. [21], suggesting migrant Men who have Sex with Men (MSM) or money boys^1^ had sex without a condom because they were under the influence of alcohol. However no statistical tests were performed.

Among stationary migrants, there was a general trend towards a clear-cut association between alcohol consumption and unprotected sex [22, 23]. However, studies looking into migrant MSM did not come to this conclusion. Alcohol intoxication was not significantly associated with condom use/non-use among migrant MSM [24], and when the comparison was made between migrant MSM and general male migrant populations, there was no statistical difference in terms of condom use [25] (Table 1).

**Table 1:** Alcohol use vs condom use

Authors	Sample	Alcohol patterns measurements	Alcohol-related sexual risks	Association: alcohol use and sexual risk
Kissinger et al. [17]	180 male Latino immigrant workers Mean age: 33 Cross-sectional/global association study USA	Frequency of alcohol use (last week) Binge drinking (consuming more than 4 drinks in one sitting)	Inconsistent condom use, Multiple sexual partners, paid sex	Migrant binge drinkers were more likely not to have used condom in their last sexual intercourse, in week before the survey (40 vs 21 % OR 2.11 CI 1.06–4.20, p < 0.05), compared to not binge drinkers
Zaller et al. [19]	358 female migrants working in entertainment venues Mean age: 23.5 Cross-sectional/global association study China	AUDIT >8 (problem drinking); >16 (probable dependence) (quantity of alcohol use)	Condom use (previous 6 months)	Female migrants working in entrainment Venus were more likely to have never used a condom during vaginal sex in the previous 6 months (OR 1.89 CI 1.12–3.11). Additionally, they had been drunk before their last sexual encounter (OR 2.24, CI 1.07–4.65), compared to less intoxicated women
Ford and Chamarathrithirong [18]	3426 Male migrant workers Age: 15–49 years Cross-sectional/situational study Thailand	Alcohol use before sex	Condom use	Migrants who reported drinking alcohol with sex workers were less likely to always use a condom (OR 0.67, CI 0.52–0.85, p < 0.001) than those who did not drink alcohol
Verma et al. [16]	7602 Male migrant workers, migrant FSWs, clients of FSWs Mean age: 26.5 (male migrant Mean age: 30 (FSWs) Mean age: 26.5 (clients of FSWs Cross-sectional/situational study India (Asia)	Alcohol use prior to sex (last month) Type of alcohol use in place of destination (last month)	Number, type of sexual partners sex, multiple partners, paid sex condom use	This study found that inconsistent condom use with paid partners was significantly higher if the migrants workers, FSWs and clients of FSWs consumed alcohol prior to sex than when they did not (19 vs 39 % OR 2.7, CI 2.0–3.5, p < 0.001); (24 vs 43 % OR 3.0, CI 2.3–3.9, p < 0.001) and (24 vs 43 % OR 2.7, CI 2.0–3.5, p < 0.001), respectively Inconsistent condom use among migrants male workers, FSWs and clients of FSWs with non-paid/casual sex partners were both significantly higher if migrants consumed alcohol prior to sex than when they did not (80 vs 79 % OR 0.9, CI 0.7–1.3, p < 0.001); (46 vs 57 % OR 1.5, CI 1.1–2.0, p < 0.001) and (55 vs 74 %, OR 2.7, CI 1.8–4.1) respectively
Sanchez [35]	278 male Latino immigrant workers Mean age: 37 Cross-sectional/global association study USA Florida	Frequency and quantity of alcohol, AUDIT, Unprotected sex under influence of alcohol in the past 30 days	Unprotected sex	This study revealed that migrants living in migrant camps were more likely to report unprotected sex while under influence of alcohol (OR 7.53 CI 4.31–13.09), compared to migrants who lived on their own or in someone else’s house
Organista and Kubo [20]	102 male Mexican/Latino immigrant workers Mean age: 31 Cross-sectional/global association study USA	Quantity e frequency of alcohol use, (last 6 months)	Unprotected sex	This study did not find any significant associations between alcohol consumption and unprotected sex with FSWs (p < 0.052)
Wong et al. [21]	239 male sex workers, rural to urban migrants Mean age: 25 Cross-sectional/global study China	Quantity of alcohol use (intoxication)	Condom use	Thirty four percent of male sex workers answered “yes” to the question “Have you ever had sex without a condom because you were under the influence of alcohol?” However no statistical tests were performed for this result so it is not possible to compare if condom use was more than when not under the influence of alcohol
Rojas et al. [22]	527 male Latino immigrants Age: 18–39 Based in Longitudinal study (3 years) data collection (Global association study) USA	Frequency and quantity of alcohol use; AUDIT (3 months prior to immigration sexual risk behaviour)	Unprotected sex	Findings of this study showed during pre-immigration, higher levels of alcohol use was associated with lower a lower frequency of condom use (ß = −0.15, p < 0.01), more sexual partners (b = 5.28, p < 0.001) and more recurrent exchanges of sex for money or drugs (b = 5.30, p < 0.001), as well as more sexual acts under the influence of alcohol (b = 5.52, p < 0.001)
Rao et al. [23]	4595 Male migrants Age: 15–49 Cross-sectional/global study India	Frequency of alcohol use	Inconsistent condom use	Migrants who regularly consume alcohol were significantly more likely to have had unprotected sex with FSWs, than those who do not consume alcohol (29 vs 5 % OR 7.87 CI 4.62–13.40, p < 0.001)
Liu et al. [24]	307 male migrant MSM Mean age: 24 Cross-sectional/global study China	Frequency of alcohol use	Condom use	The study concluded that alcohol intoxication was not significantly associated with condom use/non-use among migrant MSM
He et al. [25]	239 male migrant MSM-money boys and 100 general male migrant Mean age MSM: 25 Mean age: 30 Cross-sectional/ situational study China	Alcohol use before sex	Unprotected sex with casual partners	It was observed there was no statistical difference in terms of condom use under influence of alcohol, 34 % of participants from both groups reported having unprotected sex while under influence of alcohol (p = 0.347). However, migrant MSM presents the possibility for more risky sexual behaviour. For instance, MSM are more likely to have casual sex or one-night stands (82 vs 34 % p < 0.001) compared to general male migrant

### Alcohol use and type of sexual partners (paid, non-paid/casual partners), and premarital sex

Results among male and female migrant labourers consistently showed a positive association between high consumption of alcohol and engaging in high risk sexual behaviour with numerous types of sexual partners [26, 27]. Migrants who consume alcohol are more prone to engage in sex with any type of partners (paid/non-paid and casual partners) [10, 16, 28–30].

The population of migrant FSWs also showed a significant relation between alcohol use and sex with three or more casual sex partners [16]. The same result was found among stationary migrants [31, 32] (Table 2).

**Table 2:** Alcohol use vs MSP, type of sexual partners (paid, non-paid/casual partners), and premarital sex

Authors	Sample	Alcohol patterns measurements	Alcohol-related sexual risks	Association: alcohol use and sexual risk
Weine et al. [26]	400 male labour migrants Mean age: 32.8 Cross-sectional/global association study Russia	Heavy alcohol drinks (>3/week)	Number and type of sexual partners	Migrants that have more vaginal sex with women other than their steady sexual partners in the previous month had a significantly positive association with heavy alcohol drinking—imbibing more than three times per week (b = 0.24, p < 0.004)
Amirkhanian et al. [27]	499 Male labour migrants Mean age: 31.9 Cross-sectional/global association study Russia	Quantity and frequency of alcohol use	Total number of female sexual partners in the past year and in the past 3 months	This study revealed that the number of casual female partners over a 3 month period was considerably linked to alcohol use (b = 0.36 p < 0.004) and paid sex (OR 1.05 CI 1.02–1.09 p < 0.001)
Kissinger et al. [17]	180 male Latino immigrant workers Mean age: 33 Cross-sectional/global association study USA	Frequency of alcohol use (last week) Binge drinking (consuming more than four drinks in one sitting)	Inconsistent condom use, multiple sexual partners, paid sex	Migrant binge drinkers are more likely to have high risk partners (FSWs), than those who did not drink large quantities of alcohol (74 vs 60 %, OR 2.20 CI 1.25–5.19, p < 0.05)
Verma et al. [16]	7602 Male migrant workers, migrant FSWs and migrant clients of FSWs Mean age: 26.5 (Male migrant. Mean age: 30 (FSWs) Mean age: 26.5 (clients of FSWs Cross-sectional/situational study India (Asia)	Alcohol use prior to sex (last month) Type of alcohol use in place of destination (last month)	Number, type of sexual partners sex, multiple partners, paid sex condom use	The proportion of male migrant workers who reported sex with three or more partners was significantly higher among alcohol users than among non-users (13 vs 3 % OR 5.1, CI 4.0–6.5, p < 0.001) Sex with any partner in the previous 12 months, was significantly higher for those migrants who consumed alcohol than for those who did not (32 vs 10 %, OR 5.0, CI 4.3–5.8, p < 0.001) The same result applied to those who had sex with both paid and non-paid/casual partners (9 vs 25 %, OR 6.5, CI 4.6–9.1, p < 0.001) Male migrants who drink alcohol are more prone to engage in sex with paid, non-paid/casual partners than those who do not drink (47 vs 29 %, OR 1.9, CI 1.2–2.8, p < 0.01) Migrant FSWs who drink alcohol are more likely to report sex with three or more sexual partners than those who do not drink (68 vs 63 %, OR 1.2, CI 1.1–1.5, p < 0.001) However, migrants who had sex with both paid, not-paid/casual partners showed a higher ratio among drinkers when compared to non-drinkers—(33 vs 24 %, OR 1.5, CI 1.3–1.8, p < 0.001)
Gupta et al. [10]	174.365 male permanent and temporary migrants Mean Age: 31 Cross-sectional/global association study India	Frequency of alcohol use	Number and type of sexual partners; transactional sex; unprotected sex (Last 12 months), STI	Circular migrants—away from home for more than 1 month—who drink alcohol almost daily, are more likely to have two or more sexual partners, (12 vs 3 %, p < 0.001). They are more prone to have high risk sexual intercourse (14 vs 4 %, p < 0.001), and resort to paid sex (5 vs 3 %, p < 0.001) than those who are not mobile
Roy et al. [29]	437 male rural to urban migrant taxi drivers Mean age: 28.3 Cross-sectional/global association study Bangladesh	Frequency of alcohol use	Patterns of sexual intercourse, types and numbers of sexual partners and condom use patterns	Migrants had higher odds of risky sexual behaviour such as patterns of sexual intercourse, types and number of sex partners, and condom use patterns—if they regularly drank alcohol (OR 8.7, CI 4.9–14.3, p < 0.001)
Tiruneh [28]	756 male seasonal migrants Mean age: 22 Cross-sectional/situational study Ethiopia	Alcohol use at last sexual intercourse	Multiple sexual partners	Seasonal workers who consumed alcohol before sexual intercourse were 1.69 times more likely to have multiple sexual partners (≥2), during the preceding 6 months, as compared to those who did not drink alcohol (OR 1.69, CI 1.01–2.83, p < 0.046)
Althoff et al. [31]	113 male Latino immigrant men Mean age: 32.2 Longitudinal/global study USA	Binge drinking (having five or more drinks in one sitting)	Multiple Sexual partnerships	Binge drinking was one of the primary predictors of multiple sexual partnership (OR 2.02 CI 1.22–3.35 p < 0.01)
Wilson et al. [32]	128 Male Mexican immigrants Age: 18-60 Cross-sectional study/global studies USA	Frequency of alcohol use	Sex with FSWs and STIs	This study revealed slightly more than 14 % of the men consumed alcohol 5 or more days per week and they reported to have sex with commercial sex worker in the year preceding the study (OR 35.8, CI 3.58–333.36, p < 0.002)
Lin et al. [30]	2.153 male young rural–urban migrants Age: 18–30 Cross-sectional/global association study China	Frequency and quantity of alcohol use, alcohol intoxication (last month)	Premarital sex, number of sexual partners, transactional sex, transactional sex	This study demonstrated that overall, intoxicated respondents were more likely to engage in premarital sex than those who had not been drinking (76 vs 60.2 %, OR 1.30, p < 0.001); to have multiple sexual partners (14 vs 5 %, OR 1.57, p < 0.001); to buy sex (13 vs 5 %, OR 1.88, p < 0.001); and to sell sex (10 vs 4 %, OR 1.99, p < 0.001). No significant difference was found between men and women
Chen et al. [7]	3026 male and female (rural migrants and rural and urban residents) Age: 18–40 Cross-sectional/global study China	Frequency of alcohol use (intoxication)	Commercial sex	This showed the rural male migrant were more likely to engage in alcohol intoxication combined with commercial sex, than female rural migrant (9 vs 2 %). However, rural male migrants were less likely to combine alcohol intoxication to commercial sex, when compared to the non-migrant rural and urban residents (9 vs 9.8 vs 13 %, p < 0.05)

### Alcohol use and STI symptoms and HIV infections

The three studies focusing upon migrants confirmed the positive association between alcohol consumption and STI and HIV infections [32–34] (Table 3).

**Table 3:** Alcohol use, STI symptoms and HIV infections

Authors	Sample	Alcohol patterns measurements	Alcohol-related sexual risks	Association: alcohol use and sexual risk
Surrat [33]	101 Female migrant sex workers Mean age: 31 Cross-sectional/global association study USA Virgin Island	Life time alcohol use	STI	Migrant female workers also disclosed alcohol consumers were much more likely to report current STI symptoms than non-drinkers (OR 4.97 CI 1.51–16.20 p < 0.01)
Zuma et al. [34]	835 Female circular migrant women Cross-sectional/global association study South Africa	Frequency of alcohol use	HIV prevalence	Findings of this study showed that drinking alcohol at least once a day over a 4 week period was associated with HIV prevalence (OR 1.92 CI 1.57–3.19) p < 0.011)
Nichols et al. [36]	Predominantly Male work migrants; N = 9 neighborhoods in the town Ecological study Namibia	Visiting of registered and unregistered drinking establishments	HIV infection	Increased prevalence of HIV was observed in neighbourhoods with high density of drinking establishments compared to those with a low density. This connection was even stronger when there was a high prevalence of unregistered drinking establishments known as shebeens (OR 3.02, CI 2.04-4.47; OR 1.71, CI 1.4; OR 1.55, CI 1.19–2.02)
Wilson et al. [32]	128 male Mexican immigrants Age: 18–60 Cross-sectional study/global studies USA	Frequency of alcohol use	Sex with FSWs and STIs	Immigrant Latino men who consumed alcohol 5 or more days per week had higher odds of reporting STIs (38 vs. 7.1 %, OR 7.8 CI 2.19–27.80, p < 0.002) than those who consumed alcohol less than 5 days a week

### Predictors of alcohol use and risky sexual behaviour

This review identified individual factors such as psychological and behavioural characteristics as predictors of alcohol-related sexual risks. For instance, prior to visiting FSWs, men drink to feel more relaxed [20]. Male migrants who engaged in sex with FSWs were more likely to drink alcohol than those who did not [19].

Furthermore, socio-demographic factors, such as younger age, place of residence, and lack of education are also predictors of alcohol use and risky sexual behaviour [27]. For example, less educated migrant FSWs were more prone to engage in alcohol and unprotected sex [33].

Apart from this, environmental factors also seem to be predictors of alcohol-related sexual risks. Weine et al. [26] suggested working and living conditions tend to amplify masculine norms associated with alcohol-related sexual risks. This includes unprotected sex under influence of alcohol [35]. Moreover, Zaller [19] revealed women working in entertainment venues are more prone to engage in alcohol-related risks than those who do not work in such environments. This occurs independently of them being FSWs or not.

Additionally, increased HIV prevalence was observed in neighbourhoods with a high density of drinking establishments. This connection grew even stronger when there was a higher prevalence of unregistered establishments such as shebeens [36].

Our study also revealed male migrants with a high degree of mobility are more prone to engage in alcohol-related sexual risks than those who were less mobile. However, among the sub-population of migrant FSWs, high mobility indicated only a minor connection with alcohol-related sexual risk activities [10, 16].

Sex workers are considered to be a high-risk group. However, FSWs and their clients as well as MSM money boys, seem not to engage in higher alcohol-related sexual activities than the general migrant population. For instance, high mobility male migrants were more prone to use alcohol prior to sex (p < 0.001), than migrant FSWs (p < 0.05); and their clients [16]. Additionally, the study by He et al. [25] did not find any statistical differences between MMS money boys and the general migrant male population.

Finally, we identified that male migrants are more prone to engage in alcohol-related risks than female migrants [7].

## Discussion

We based our analysis on global studies, in which subjects’ general patterns of alcohol use are examined in relationship to their typical patterns of sexual behaviour, and that among different types of migrant populations. Our review disclosed the highest odds of reporting risky sexual behaviour such as unprotected sex with FSWs and STIs is among migrants who consumed alcohol frequently and/or heavily [23, 32].

Practices related to sexual intercourse, types and number of sex partners, and patterns of condom use were observed more frequently with migrants who reported drinking alcohol than with those that did not [29]. Studies examining the overall relationship between drinking status and sexual behaviour are limited to describing patterns of general covariation. They do not provide information about the degree to which alcohol use and sexual behaviour occur on the same occasion. Therefore, as such, they cannot inform us about potential causal effects of alcohol upon sexual behaviour [37].

We also based our analysis on situational studies. Clearly, it is important to determine whether alcohol is being consumed proximal to the act of sexual intercourse. Overall, the situational studies in this review, indicated migrants who use alcohol and/or intoxicating substances prior to or during sexual intercourse were more prone to engage in sexually risky behaviour. This included having more unprotected sex, MSP and paid sex, than those who did not drink [16, 18].

These studies are somewhat more informative than global studies, as they examine effects from the use of alcohol during sexual events. However, they still obscure the important temporal relationship between alcohol use and risky sexual behaviour. These studies still fail to describe what happens on an event-by-event basis, and thus do not determine whether the occasions characterised by unprotected intercourse were the same occasions during which alcohol was consumed [37].

Apart from that, this literature is composed mostly of cross-sectional studies relying on self-reported alcohol use and sexual behaviour. Findings are therefore constrained in terms of their ability to draw causal conclusions, and all reports of behaviour in this literature must be interpreted with caution.

Only one longitudinal study was found but what this review is most seriously missing are more longitudinally and event level designed studies of the relation between alcohol use and sexual risks. Only with prospective research can temporal associations between alcohol use and sexual risk behaviours be disentangled [38].

However, this review did suggest the link between alcohol use and risky sexual behaviour among migrant populations is an important but under-researched issue all over the world. We were only able to identify 22 articles for our review, and these were concentrated in the USA and China. However, they only focused on the receiving communities with little attention paid to the pre-immigration characteristics of migrant populations or for cultural factors that maybe account for migrants’ alcohol use and risky sexual behaviour.

We only found one study that compared pre and post migration HIV risk factors among migrant men and women [35]. It revealed post migration increases migrants’ risky sexual behaviour. Both men and women increased their alcohol-related sexual risks while they were in host countries (USA).

These reviewed studies are also limited in number and scope in relation to the measures of alcohol use and sexual risk behaviour among migrant populations. For example, we only identified three studies conducted in Sub-Saharan Africa. This region carries the world’s greatest HIV/AIDS burden, and southern Africa also consumes great quantities of alcohol [2].

Nevertheless, one relevant trend existed among all migrant populations encountered here—labourers, sex workers and stationary immigrants. There was a consistent negative association between alcohol consumption and risky sexual behaviour. Although we did not have the means to compare the different types of the migrant populations in terms of their alcohol-related sexual risks, the study by Verma et al. [16] reveals migrant FSWs and their clients were less likely to engage in alcohol-related sexual activities than male migrants.

The study by He et al. [25] did not encounter any significant difference between MSM money boys and male migrants. These results show the need for future investigation, both observational and interventional, including subpopulations of migrant men and women in diverse geographic locations, and multiple outcomes to assess the link between alcohol use and sexual risk.

Among the studies identified in this review, one rare exception delved into the differences between alcohol-related sexual risks among migrant and general populations. It revealed that rural male and female migrants were less or equally likely to report alcohol-related risks than rural and urban non-migrants [7]. Furthermore, no significant difference was encountered in comparison to Chinese migrants reviewed by Li et al. [15]. This indicates more research is required on the difference between migrant and non-migrant populations to assess factors contributing to risky sexual behaviour in migratory contexts. Otherwise, there is a risk inaccurate generalisations will be made about migrant populations, when in fact the results only apply to the general population.

## Limitations of the review

We recognise a meta-analysis study would be the best way to correlate the variables in this study. However, migration and mobility were assessed by several measurements—differing socio-economic backgrounds, ages ranging from adolescents to adult, different gender groups, and the regions where studies were conducted. Finally, global and situational measurements of alcohol use and risk behaviour were assessed.

Findings are often limited to global and situational level studies with few experimentally based examinations. Therefore in-depth examination of the specific situation where two behaviours co-occur, the social and cultural context of sexual activity was not researched in sufficient detail.

For instance, neither condom usage during intercourse nor the type of sexual partner—steady or casual—nor the amount of alcohol consumed prior to engaging in sexual activity could be assessed for each and every reported sexual encounter. Moreover, the majority of studies were hampered by their reliance on self-reporting of alcohol consumption and risky sexual behaviour, which is often influenced by social appropriateness.

Furthermore, this review lacks information regarding pre-immigration characteristics of migrant populations, as well as information regarding their communities of origin. This information would be instrumental for comparing former alcohol-related sexual risks in their home environment and the same activities in host communities. Moreover, we recognise specific cultural factors potentially account for certain associations in behavioural patterns.

In addition, the present review is limited by studies published in English and dependent upon availably in the PubMed database.

These limitations notwithstanding, the present literature review provides a broad overview of recent research on alcohol use and risky sexual behaviour among migrant/mobile populations. It also highlights the extent and consistency of significant associations among the variables measured for this type of population.

## Conclusions and implications

This review determined there is a significant and positive association between global and situational alcohol use and several outcomes of risky sexual behaviour among various types of male and female migrant populations. This reveals that migrants should be considered target groups for HIV interventions and strategies.

However, the association between alcohol use and risky sexual behaviour is not linear among the many types of migrant populations. It was mainly observed at high quantities and frequency of alcohol use, mainly in younger male migrants. As well, it was often tied to a specific situation or context. High risk sexual partners, levels of mobility, contextual and environmental factors such as living arrangements, entertainment venues are some of the variables that may increase the likelihood to engage in hazardous alcohol consumption. Thus, this increases the propensity to enter into high risk sexual behaviour. We therefore conclude it is useful to consider risk environments rather than attributing risk to types of people. Consequently, we propose the following recommendations:

Rather than focus on the traditional behavioural science approach which only takes into account individual factors, we suggest broadening it for more comprehensive HIV interventions approaches. These would include the structural, environmental, cultural, socio-relational and sexual contexts which create risk environments for migrant populations.

Employers should be encouraged to improve working conditions and provide more family-friendly housing arrangements for migrant workers. Evidence suggests poor working and living conditions amplify masculine norms associated with high risk sexual behaviour including alcohol consumption and engaging with multiple sex partners. Strengthening migrants’ capacities for alternative ways of coping with such difficulties could also be a focus of multilevel interventions. For migrant populations, for example, change could be initiated by developing new ways of socialisation including recreational activities. The goal here would be to encourage them to shift away from their traditional notions regarding alcohol use and sexual infidelity [26].

Furthermore, multilevel alcohol Venue-based HIV interventions—that is, interventions targeted at the individual and the settings, such as shebeens [38]—may be required to prevent HIV transmission among migrants. Such interventions would primarily target men who have a high degree of mobility and frequently drink large quantities of alcohol.

Combined approaches such as scientifically proven, cost-effective, and scalable interventions, including biomedical and behavioural interventions represent achievable goals. Additionally, other public health strategies—HIV testing, alcohol and substance abuse treatment programs—are important for reducing HIV transmission among migrants who engage in excessive alcohol consumption [39].

Such approaches need to involve basic research able to identify and link contextual factors to HIV risk in migrants as well as build on the personal agency and resilience of these specific populations [40]. It is also of great importance to further investigate the intersection of population, geographical space or mobile situation together with behaviours of interest, as well as incorporated cultural contexts of migrant populations.

To evaluate the impact of migration on these issues, future studies should also focus on comparing various types of migrant populations, to assess migrants with non-migrants and to provide a theoretical framework on how migration interacts with alcohol consumption and risky sexual behaviour. To measure the possible influential aspects of migration, qualitative methods should be applied when a quantitative approach is not sufficient to explore the underlying influencing factors of migration.

In addition, this review indicates research should use more event level methodologies to better understand how alcohol abuse affects the migrant population’s risk behaviour. This would provide stronger evidence and conclusions as to whether or not there is a general causal relationship between alcohol consumption and risky sexual behaviour among migrant populations.

## Footnotes

1 Men who engage in same-sex transactional sex for economic survival.
